# Prognostic factors and survival according to tumour subtype in women presenting with breast cancer bone metastases at initial diagnosis: a SEER-based study

**DOI:** 10.1186/s12885-020-07593-8

**Published:** 2020-11-13

**Authors:** Xiao Li, Xiaoli Zhang, Jie Liu, Yinzhong Shen

**Affiliations:** 1 Department of Medical Affairs, Shanghai Public Health Clinical Center, Fudan University, Shanghai, China; 2 Department of Health Statistics, School of Public Health, China Medical University, Shenyang, China; 3 Department of Infection and Immunity, Shanghai Public Health Clinical Center, Fudan University, Shanghai, China

**Keywords:** Breast cancer, Bone metastases, Tumour subtype, Prognostic factor, Survival

## Abstract

**Background:**

Tumour subtype has a significant effect on bone metastasis in breast cancer, but population-based estimates of the prognosis of patients with bone metastases at breast cancer diagnosis are lacking. The aim of this study was to analyse the influence of tumour subtype and other factors on the prognosis and survival of patients with bone metastases of breast cancer.

**Methods:**

Using the Surveillance, Epidemiology, and End Results (SEER) Program data from 2012 to 2016, a retrospective cohort study was conducted to investigate stage IV breast cancer patients with bone metastases. Stage IV patient characteristics according to subtype were compared using chi-square tests. Overall survival (OS) and prognostic factors were compared using the Kaplan-Meier method and the Cox proportional hazards model, respectively.

**Results:**

A total of 3384 stage IV patients were included in this study; 63.42% were HR+/HER2-, 19.86% were HR+/HER2+, 9.34% were HR−/HER2-, and 7.39% were HR−/HER2+. The median OS for the whole population was 38 months, and 33.9% of the patients were alive at 5 years. The median OS and five-year survival rate were significantly different among stage IV breast cancer patients with different molecular subtypes (*p* < 0.05). Multivariate Cox regression analysis showed that age of 55–59 (HR = 1.270), black race (HR = 1.317), grade III or IV (HR = 1.960), HR−/HER2- (HR = 2.808), lung metastases (HR = 1.378), liver metastases (HR = 2.085), and brain metastases (HR = 1.903) were independent risk factors for prognosis; married status (HR = 0.819), HR+/HER2+ (HR = 0.631), HR−/HER2+ (HR = 0.716), insurance (HR = 0.587) and surgery (HR = 0.504) were independent protection factors of prognosis. There was an interaction between the HR+/HER2+ subtype and other metastases (except bone metastases, HR = 0.694, 95% CI: 0.485–0.992), but the interaction between race and subtype did not reach significance for prognosis.

**Conclusions:**

There were substantial differences in OS according to tumour subtype. In addition to tumour subtype, other independent predictors of OS were age at diagnosis, race, marital status, insurance, grade, surgery and visceral metastases. There was an interaction between the HR+/HER2+ subtype and other metastases (except bone metastases) for prognosis. Tumour subtype, as a significant prognostic factor, warrants further investigation.

## Background

Breast cancer is the second most common type of cancer in women and the second leading cause of cancer-related death in women. In these patients, it is not the primary tumour but its metastases at distant sites that are the main cause of death [1]. Approximately 5–10% of patients have distant metastases at the time of diagnosis [2, 3]. Bone is the most common site of metastasis in breast cancer patients, and more than 55% of breast cancer patients develop bone metastases [4]. Bone metastases are associated with lower survival in patients with advanced breast cancer [5]. A study showed that patients with breast cancer survive a median of 22–57.6 months after the detection of bone metastases [6–8]. Breast cancer patients with bone metastases seem to have a longer survival than those with cancer in other metastatic sites [9].

According to the classification by hormone receptor status and human epidermal growth factor receptor-2 **(**HER2**)**, breast cancer can be divided into HR+/HER2-, HR+/HER2+, HR−/HER2- and HR−/HER2+ subtypes [10]. The strong association of hormone receptor status with bone metastasis was proposed in 1991 [11]. With a deeper understanding of the modulated genes and pathways in the various subgroups, it has become more evident that bone metastasis is most abundant among the hormonal receptor-positive subtypes [12]. Researchers have found that the clinical manifestations, pathological results, gene expression and prognosis of different subtypes of breast cancer are very different. The relationship between molecular subtype and the patterns of distant metastases has been documented. Evidence has shown that the risk of bone metastasis depends on the breast cancer subtype, and HR+ patients are more likely to have bone metastases [13]. The molecular differences in tumour subtype are often accompanied by differences in clinical features and overall survival [10]. The distribution of molecular subtypes is different among breast cancer patients with different races, and race is a prognostic factor of breast cancer patients [14, 15]. However, the effect of mixed race and subtype on prognosis has not been verified.

Notably, once a tumour metastasizes to the bone, it is incurable. Bone metastases are associated with lower survival in patients with advanced breast cancer and an increased risk of serious complications during the patient’s disease course. The consequences of bone metastases include reduced survival, morbidity, pain and reduced quality of life [16]. Therefore, to improve the survival time and outcome of patients, identifying the influencing factors of clinical prognosis in breast cancer patients with bone metastasis has great significance. The aim of this study was to analyse the influence of tumour subtype and other factors on the prognosis and survival of patients who present with bone metastases at the time of initial diagnosis of breast cancer.

## Methods

### Data source and patient selection

We extracted data from the Surveillance, Epidemiology, and End Results (SEER) 18 registry research database. The SEER database of the National Cancer Institute is a coordinated system of population-based cancer registries that collects cancer incidence and survival data from 18 geographic areas throughout the United States that together represent approximately 28% of the U.S. population and includes various diverse ethnic groups. A data use agreement submission was required to access the SEER Research Data File [17]. We submitted the data agreement form to the SEER administration. After acceptance of the agreement, SEER*Stat Version 8.3.5 software and data files were downloaded directly from the SEER website.

We used SEER*Stat version 8.3.5 to generate a case listing. We extracted cases of women aged 40–60 with bone metastases of breast cancer diagnosed with a known breast subtype. Because the SEER database began collecting information on the HER2 status and sites of distant metastasis in 2010, the most recent edition of The SEER Cancer Statistics Review (CSR) (1975–2016) was released in April 2019. We explored the situation of breast cancer patients in the past 5 years, including women aged 40–60 diagnosed between 2010 and 2013 and selected this age group of women because the incidence of breast cancer increases over the age of 50, the natural mortality of elderly patients is high, and age is the second most important risk factor at primary diagnosis [18].

Patients diagnosed by either autopsy or death certificate were excluded. Patients must have complete dates of survival months and the follow-up must be active. The analysis was restricted to patients with a diagnosis confirmed by histopathology, and only duct, lobular and other carcinomas based on the primary site were included (International Classification of Diseases for Oncology, Third Edition (ICD-O-3) codes 8500 to 8543). Tumour stage was registered according to the AJCC staging system sixth edition.

We generated a case listing with information on the following variables: year of diagnosis, age at diagnosis, race/ethnicity, marital status at diagnosis, grade, laterality, ICD-O-3 Hist/behav, American Joint Committee on Cancer (AJCC) stage group 6th edition, surgery primary site, bone/lung/liver/brain metastases, tumour subtype, cause-specific death classification, vital status, and survival (months). Race was classified as white, black or other. Marital status was categorized as married, single (including never married, divorced, separated, and widowed) or other. Insurance was classified as uninsured, insured (including any Medicaid, insured, and insured-no specifics) or unknown. Because stage was suggested to be the most powerful prognostic factor in other studies and in the clinic and because stage IV patients exhibit worse survival rates than stage I–III patients, we only selected stage IV patients according to the AJCC stage group 6th edition.

### Statistical analysis

Descriptive statistics were used to examine the following baseline characteristics of stage IV breast cancer patients with bone metastases: year of diagnosis, age, race/ethnicity, insurance, marital status, grade, surgery, laterality, histology, liver metastases, lung metastases, brain metastases, bone only metastases, BCSS (the time from breast cancer diagnosis to death due to breast cancer) and OS (the time from breast cancer diagnosis to death due to any cause). Age at diagnosis, race/ethnicity, insurance, marital status, grade, surgery, laterality, histology, liver metastases, lung metastases, brain metastases, and interaction terms between visceral metastases and subtype were used in the multivariate Cox model.

The variables were stratified by molecular subtype. *P*-values for comparing the frequency distributions among the subgroups were calculated using the chi-squared (*x*^2^) test. For each variable, patients with unknown data were excluded from the comparative analysis. OS was the primary study outcome, and we used the Kaplan-Meier method to generate survival curves and analysed the differences between the curves using the log-rank test. A Cox proportional hazards regression model was used to assess the independent association of several variables with OS, and interaction analysis was performed by adding interaction items to the next layer. Hazard ratio (HR) and their 95% confidence intervals (95% CIs) were estimated using the Cox model. A *P*-value of 0.05 or less was considered statistically significant. All *P*-values were 2-tailed. All statistical analyses were performed using SAS version 9.2 (SAS Institute, Inc.) and IBM SPSS version 23.0.

## Results

### Patient characteristics

A total of 3384 stage IV patients were diagnosed with bone metastases from breast cancer at initial presentation between 2012 and 2016 and were included in this study. A total of 2146 stage IV patients (63.41%) were diagnosed with HR+/HER2- breast cancer, 672 stage IV patients (19.86%) were diagnosed with HR+/HER2+ breast cancer, 316 stage IV patients (9.34%) were diagnosed with HR−/HER2- breast cancer, and 250 stage IV patients (7.39%) were diagnosed with HR−/HER2+ breast cancer.

The demographic and clinical characteristics of the study participants based on breast cancer subtype are shown in Table 1. Stage IV patients with bone metastases from HR−/HER2- breast cancer were more likely to be white (*P* < 0.001). Patients with HR−/HER2-, HR+/HER2+ and HR−/HER2+ breast cancers were more likely to have higher tumour grades and a histological type classification of ductal carcinoma than those with HR+/HER2- breast cancer (*P* < 0.001). Visceral and only bone metastases were less frequent in HR+/HER2- breast cancer patients (*P* < 0.001). HR−/HER2- breast cancer patients were more likely to die (*P* < 0.001).

**Table 1:** Patient Characteristics According to Tumour Subtype

Characteristics	HR+/HER2-	HR+/HER2+	HR−/HER2-	HR−/HER2+	Total	*P-*value
	2146(63.41)	672(19.86)	316(9.34)	250(7.39)	3384(100)	
Year of diagnosis						0.981
2012	422(19.66)	133(19.79)	55(17.41)	51(20.40)	661(19.53)	
2013	441(20.55)	138(20.54)	68(21.52)	52(20.80)	699(20.66)	
2014	425(19.80)	125(18.60)	65(20.57)	46(18.40)	661(19.53)	
2015	428(19.94)	143(21.28)	59(18.67)	45(18.00)	675(19.95)	
2016	430(20.04)	133(19.79)	69(21.84)	56(22.40)	688(20.33)	
Age at diagnosis						0.221
40–44 years	283(13.19)	107(15.92)	31(9.81)	28(11.20)	449(13.27)	
45–49 years	465(21.67)	128(19.05)	65(20.57)	50(20.00)	708(20.92)	
50–54 years	638(29.73)	202(30.06)	110(34.81)	81(32.40)	1031(30.47)	
55–59 years	760(35.41)	235(34.97)	110(34.81)	91(36.40)	1196(35.34)	
Race						< 0.001
White	1574(73.35)	481(71.58)	218(68.99)	170(68.00)	2443(72.19)	
Black	353(16.45)	127(18.90)	81(25.63)	43(17.20)	604(17.85)	
Other ^a^	211(9.83)	64(9.52)	16(5.06)	33(13.20)	324(9.57)	
Unknown	8(0.37)	0(0.00)	1(0.32)	4(1.60)	13(0.38)	
Marital status						0.771
Single	620(28.89)	189(28.13)	89(28.16)	73(29.20)	971(28.69)	
Married	1049(48.88)	342(50.89)	150(47.47)	113(45.20)	1654(48.88)	
Other ^b^	377(17.57)	111(16.52)	64(20.25)	47(18.80)	599(17.70)	
Unknown	100(4.66)	30(4.46)	13(4.11)	17(6.80)	160(4.73)	
Insurance						0.490
Insured	104(4.85)	42(6.25)	17(5.38)	11(4.40)	174(5.14)	
Uninsured	2027(94.45)	623(92.71)	297(93.99)	235(94.00)	3182(94.03)	
Unknown	15(0.70)	7(1.04)	2(0.63)	4(1.60)	28(0.83)	
Grade						< 0.001
I	247(11.51)	21(3.13)	4(1.27)	2(0.80)	274(8.10)	
II	1017(47.39)	244(36.31)	64(20.25)	67(26.80)	1392(41.13)	
III or IV	619(28.84)	334(49.70)	230(72.78)	149(59.60)	1332(39.36)	
Unknown	263(12.26)	73(10.86)	18(5.70)	32(12.80)	32(12.80)	
Histology						< 0.001
Ductal	1571(73.21)	559(83.18)	277(87.66)	216(86.40)	2623(77.51)	
Lobular	353(16.45)	28(4.17)	13(4.11)	6(2.40)	400(11.82)	
Others	222(10.34)	85(12.65)	26(8.23)	28(11.20)	361(10.67)	
Laterality						0.153
Right	1054(49.11)	330(49.11)	156(49.37)	102(40.80)	1642(48.52)	
Left	1070(49.86)	337(50.15)	155(49.05)	146(58.40)	1708(50.47)	
Bilateral, single primary	5(0.23)	4(0.60)	2(0.63)	1(0.40)	12(0.35)	
Unknown	17(0.79)	1(0.15)	3(0.95)	1(0.40)	22(0.65)	
Lung metastases						< 0.001
No	1643(76.56)	452(67.26)	216(68.35)	158(63.20)	2469(72.96)	
Yes	449(20.92)	205(30.51)	96(30.38)	89(35.60)	839(24.79)	
Unknown	54(2.52)	15(2.23)	4(1.27)	3(1.20)	76(2.25)	
Liver metastases						< 0.001
No	1691(78.80)	418(62.20)	208(65.82)	132(52.80)	2449(72.37)	
Yes	423(19.71)	244(36.31)	101(31.96)	155(46.00)	883(26.09)	
Unknown	32(1.49)	10(1.49)	7(2.22)	3(1.20)	52(1.54)	
Brain metastases						< 0.001
No	1992(92.82)	595(88.54)	263(83.23)	214(85.60)	3064(90.54)	
Yes	107(4.99)	62(9.23)	44(13.92)	31(12.40)	244(7.21)	
Unknown	47(2.19)	15(2.23)	9(2.85)	5(2.00)	76(2.25)	
Only bone metastases						< 0.001
No	748(34.86)	360(53.57)	170(53.80)	168(67.20)	1446(42.73)	
Yes	1326(61.79)	300(44.64)	140(44.30)	80(32.00)	1846(54.55)	
Unknown	72(3.36)	12(1.79)	6(1.90)	2(0.80)	21(2.72)	
Surgery						0.971
No	1541(71.81)	489(72.77)	225(71.20)	180(72.00)	2435(71.96)	
Yes	588(27.40)	179(26.64)	88(27.85)	68(27.20)	923(27.28)	
Unknown	17(0.79)	4(0.60)	3(0.95)	2(0.80)	26(0.77)	
Breast cancer -specific death						< 0.001
No	1502(69.99)	504(75.00)	140(44.30)	165(66.00)	2311(68.29)	
Yes	644(30.01)	168(25.00)	176(55.70)	85(34.00)	1073(31.71)	
Status						< 0.001
Alive	1350(62.91)	474(70.54)	92(29.11)	153(61.20)	2069(61.14)	
Dead	796(37.09)	198(29.46)	224(70.89)	97(38.80)	1315(38.86)	

Other ^a^ (American Indian/AK Native, Asian/Pacific Islander)

Other ^b^ (divorced/widowed/separated)

Patients with unknown data were excluded from the comparative analysis.

### Survival analysis

At a median follow-up of 17 months (range, 1–60 months), there were 1315 deaths (60.53% in the HR+/HER2- group, 15.06% in the HR+/HER2+ group, 17.03% in the HR−/HER2- group and 7.38% in the HR−/HER2+ group).

**Fig. 1:**
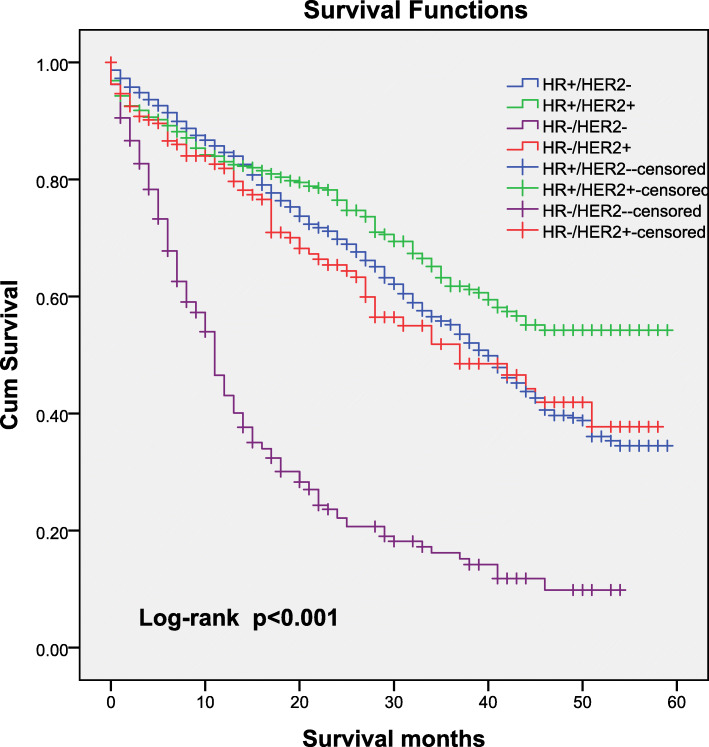
Kaplan–Meier curve for overall survival according to tumour subtype

The median OS for the entire population was 38 months (95% CI: 35.89–40.11 months), and 33.9% of the patients (95% CI: 30.6–37.2%) were alive at 60 months. Analysis of OS according to tumour subtype showed significant differences among stage IV patients with bone metastases, and the five-year survival rate was 32.7% for HR+/HER2- patients, 48.8% for HR+/HER2+ patients, 8.6% for HR−/HER2- patients and 36.1% for HR−/HER2+ patients. Stage IV HR−/HER2- breast cancer patients with bone metastases experienced the shortest survival (median OS: 11 months; 95% CI: 9.9–12.1 months), whereas stage IV HR+/HER2+ breast cancer patients with bone metastases experienced the longest survival, with a median OS of 52 months (95% CI was not estimable; *P* < 0.001). Shown in Fig. 1.

**Fig. 2:**
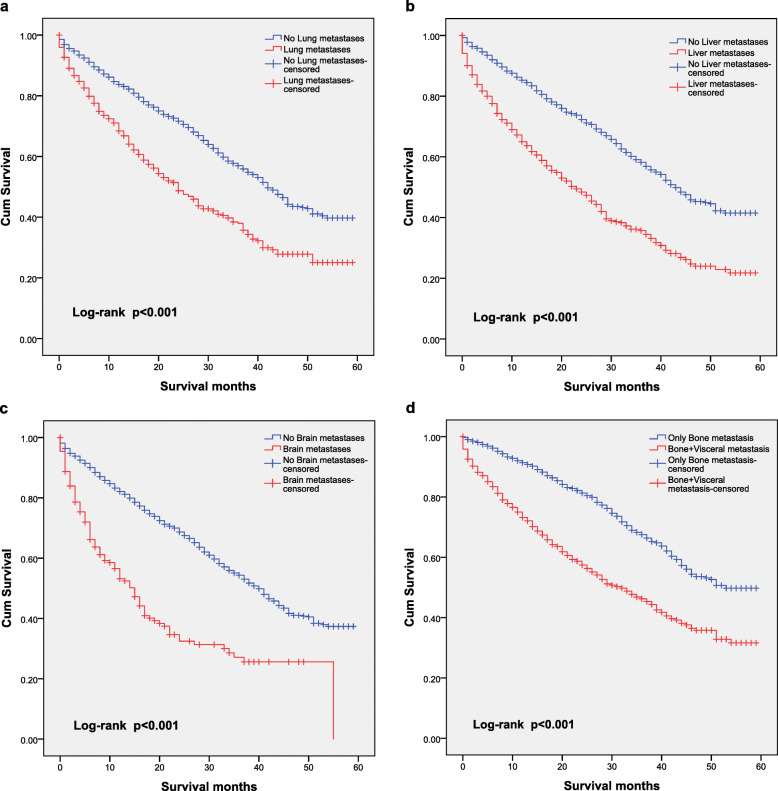
Kaplan–Meier curves for overall survival according to the metastasis site

The impact of the presence of metastases at each individual site on OS is shown in Fig. 2. Stage IV patients with lung metastases (median OS: 23 months; 95% CI: 19.98–26.02 months) had significantly shorter survival times than stage IV patients without lung metastases (median OS: 42 months; 95% CI: 39.77–44.23 months; *P* < 0.001; Fig. 2a). Stage IV patients with liver metastases (median OS: 22 months; 95% CI: 19.10–24.86 months) had significantly shorter survival times than those without liver metastases (median OS: 44 months; 95% CI: 41.14–46.86 months; *P* < 0.001; Fig. 2b). Stage IV patients with brain metastases (median OS: 14 months; 95% CI: 11.08–40.10 months) had significantly shorter survival times than those without brain metastases (median OS: 40 months; 95% CI: 37.80–42.20 months; *P* < 0.001; Fig. 2c). A similar finding was seen for only bone metastases (median OS: 46 months; 95% CI: 42.56–49.44 months) compared with metastases to the bone and other sites (median OS: 24 months; 95% CI: 24.61–26.38 months *P* < 0.001; Fig. 2d).

The unadjusted model results for the overall patient population were consistent with the log-rank analysis results (except laterality) and revealed that patients who were older, were black, were single, were uninsured, had ductal histology, had grade III or IV breast cancer, had primary bilateral breast cancer, had the triple-negative subtype, had visceral metastases and did not undergo surgery for the primary tumour had shorter OS (Table 2).

**Table 2:** Univariate analysis of prognostic factors

Characteristics	Median OS	*P* value	HR	95% CI for HR
Age at diagnosis		< 0.001		
40–44 years	42		Reference	
45–49 years	41		1.013	0.829–1.237
50–54 years	39		1.103	0.915–1.329
55–59 years	32		1.364	1.139–1.634
Race		< 0.001		
White	41		Reference	
Black	28		1.498	1.315–1.707
Other ^a^	38		1.003	0.824–1.220
Marital status		< 0.001		
Single	32		Reference	
Married	42		0.723	0.638–0.819
Other ^b^	35		0.903	0.772–1.057
Insurance		< 0.001		
Uninsured	26		Reference	
Insured	38		0.633	0.513–0.781
Grade		< 0.001		
I	48		Reference	
II	44		1.214	0.953–1.547
III or IV	28		2.071	1.634–2.625
Histology		0.003		
Ductal	36		Reference	
Lobular	44		0.748	0.624–0.897
Others	40		0.856	0.713–1.028
Laterality		0.084		
Right	38		Reference	
Left	38		1.058	0.949–1.179
Bilateral, single primary	13		2.254	1.008–5.039
Tumour subtype		< 0.001		
HR+/HER2-	39		Reference	
HR+/HER2+	52		0.747	0.640–0.873
HR−/HER2-	11		3.571	3.071–4.152
HR−/HER2+	35		1.132	0.917–1.397
Bone+Lung metastases		< 0.001		
No	42		Reference	
Yes	23		1.888	1.679–2.123
Bone+Liver metastases		< 0.001		
No	44		Reference	
Yes	22		2.182	1.950–2.443
Bone+Brain metastases		< 0.001		
No	40		Reference	
Yes	14		2.674	2.245–3.184
Only bone metastases		< 0.001		
No	46		Reference	
Yes	24		2.29	2.04–2.559
Surgery		< 0.001		
No	32		Reference	
Yes	52		0.496	0.433–0.568

Other ^a^ (American Indian/AK Native, Asian/Pacific Islander)

Other ^b^ (divorced/widowed/separated).

Multivariate Cox analyses confirmed that age of 55–59 (vs. age of 40–44, HR = 1.270, 95% CI: 1.032–1.562), black race (vs. white race, HR = 1.317, 95% CI: 1.127–1.540), grade III or IV (vs. grade I, HR = 1.960, 95% CI: 1.491–2.577), HR−/HER2- (vs. HR+/HER2-, HR = 2.808, 95% CI: 2.169–3.634), lung metastases (vs. no lung metastases, HR = 1.378, 95% CI: 1.188–1.598), liver metastases (vs. no liver metastases, HR = 2.085, 95% CI: 1.795–2.422), brain metastases (vs. no brain metastases, HR = 1.930, 95% CI: 1.542–2.248) were independent risk factors for prognosis; married status (vs. single status, HR = 0.819, 95% CI: 0.707–0.949), insurance (vs. no insurance, HR = 0.587, 95% CI: 0.459–0.751) and surgery (vs. no surgery, HR = 0.504, 95% CI: 0.431–0.590) were independent protection factors of prognosis. There was an interaction between the HR+/HER2+ subtype and multi-metastases (bone +visceral metastases, HR = 0.694, 95% CI: 0.485–0.992) on prognosis. Histology, primary laterality, and the interaction between race and subtype did not reach significance with this test. The multivariate Cox model is shown in Table 3.

**Table 3:** Multivariate analysis of prognostic factors

Characteristics	*P* value	HR	95% CI for HR
Age at diagnosis			
40–44 years		Reference	
45–49 years	0.542	0.932	0.741–1.170
50–54 years	0.878	1.017	0.821–1.260
55–59 years	0.024	1.270	1.032–1.562
Race			
White		Reference	
Black	0.001	1.317	1.127–1.540
Other ^a^	0.128	1.192	0.951–1.495
Marital status			
Single		Reference	
Married	0.008	0.819	0.707–0.949
Other ^b^	0.130	0.871	0.728–1.042
Insurance (yes vs no)	< 0.001	0.587	0.459–0.751
Histology			
Ductal		Reference	
Lobular	0.744	1.041	0.820–1.321
Others	0.356	1.105	0.894–1.365
Laterality			
Right		Reference	
Left	0.506	1.043	0.921–1.182
Bilateral, single primary	0.177	2.21	0.894–1.365
Grade			
I		Reference	
II	0.309	1.147	0.881–1.492
III or IV	< 0.001	1.960	1.491–2.577
Tumour subtype			
HR+/HER2-		Reference	
HR+/HER2+	0.002	0.631	0.474–0.839
HR−/HER2-	< 0.001	2.808	2.169–3.634
HR−/HER2+	0.258	0.716	0.401–1.277
Site of metastases			
Lung (yes vs no)	0.020	1.378	1.188–1.598
Liver (yes vs no)	< 0.001	2.085	1.795–2.422
Brain (yes vs no)	< 0.001	1.903	1.542–2.248
Surgery (yes vs no)	< 0.001	0.504	0.431–0.590
HR+/HER2- * multi-metastases		Reference	
HR+/HER2+ * multi-metastases	0.045	0.694	0.485–0.992
HR−/HER2- * multi-metastases	0.717	0.941	0.675–1.310
HR−/HER2+ * multi-metastases	0.564	0.828	0.437–1.572
**Next step**			
White*HR+/HER2-		Reference	
Black*HR+/HER2+	0.366	0.821	0.536–1.258
Other a*HR+/HER2+	0.652	0.862	0.453–1.641
Black*HR−/HER2-	0.323	0.817	0.546–1.220
Other a*HR−/HER2-	0.299	1.508	0.695–3.271
Black*HR−/HER2+	0.696	0.881	0.466–1.664
Other a*HR−/HER2+	0.368	0.701	0.323–1.521

Other ^a^ (American Indian/AK Native, Asian/Pacific Islander)

Other ^b^ (divorced/widowed/separated).

## Discussion

Bone metastases are the most common distant metastatic site in breast cancer, and severe complications, low quality of life, poor prognosis and significantly decreased survival rates are often associated with the occurrence of bone metastases [16]. Our study analysed recently available data on the subtypes of stage IV patients with bone metastatic breast cancer from the SEER registries in an attempt to analyse differences in the effects of the breast cancer subtype and other factors on patient prognosis.

Bone metastasis is most abundant among the HR+ subtypes, and the distribution of tumour subtypes in stage IV patients in our study was similar to that in other studies in the published literature [12, 19, 20]. Our study identified that stage IV patients with HR+/HER2- breast cancer are the most prone to bone metastasis, followed by those with HR+/HER2+ breast cancer. HR−/HER2- breast cancer has a particular propensity to metastasize to the brain and lung; brain metastasis is more common in this subtype than in the other subtypes, and bone metastasis is relatively less likely to occur, which is consistent with the findings of previous research [21]. This may be due to the different molecular subtypes of breast cancer leading to different metastasis sites due to their special molecular biological characteristics.

The median OS for the entire cohort was 38 months, which is similar to that in Kuchuk’s study, which reviewed 294 electronic records of metastatic breast cancer patients and found that the median OS from bone metastasis diagnosis was 40 months in bone metastasis patients [22]. The median OS was 46 months for stage IV patients with only bone metastases and 24 months for those with bone and other site metastases in our study, which was similar to the survival reported by previous authors in recent years [8]. A study of 815 patients with de novo or recurrent metastatic breast cancer identified that patients with visceral metastases and those with multiple metastatic sites had worse OS, findings consistent with our results [23]. The five-year survival rate was 33.9%, which is similar to that in previous studies, which showed that 24–39% of patients lived 5 years after the diagnosis of bone metastases [5]. This may be because the subjects of this study were menopausal women, while there was no limitation on the age of the previous study subjects; moreover, the proportion of elderly patients was large and their prognosis was poor, and with the improvement of treatment methods in recent years, the prognosis of patients has been improved.

Our study showed that the five-year survival rate of HR+/HER2+ stage IV patients was the highest, reaching 5.6 times that of HR−/HER2- patients. Stage IV patients with HR+/HER2+ breast cancer had the longest median survival period. Although our study showed that the incidence of bone metastasis in HR−/HER2- breast cancer patients was low, stage IV patients with HR−/HER2- tumours had the worst prognosis. With the shortest median survival time, the OS of stage IV patients with HR−/HER2- breast cancer was significantly lower than that of stage IV patients with other molecular subtypes. The large difference in prognosis observed across all tumour subtypes confirms that breast cancer is a heterogeneous disease, even in the specific group of patients with bone metastases. The improvements in OS seen in HER2+ patients could be explained in part by the efficacy of HER2-targeted agents. In Dawood’s large-scale, randomized study of 2019 women with metastatic breast cancer, HER2+ patients who received trastuzumab had an improved prognosis compared with HER2- patients [24]. However, HR−/HER2- is an invasive subtype, with the characteristics of rapid progression, strong aggressiveness, a high degree of malignancy, easy occurrence of distant metastasis, and rapid relapse [25–27]. Therefore, our study included tumour subtype as a prognostic factor and provided evidence of a clear association of age, race, marital status, insurance, tumour grade, histology, subtype, and visceral metastases in bone metastasis patients with OS. This was similar to the findings of a previous study. The Denmark data were from population-based health registries that included all women diagnosed during 1999–2011 with regional or stage II/III breast cancer and showed predictors of recurrence, metastases, and mortality, including age, hormone receptor status, and stage at diagnosis [28]. Ahn’s study showed that ER-negative status and bone metastasis combined with visceral metastasis are risk factors for OS [8]. Iqbal J’s study showed that in US women diagnosed with invasive breast cancer, survival varies by race and ethnicity, and black women are more likely to die from breast cancer within 7 years than non-Hispanic white or Asian women [29]. A previous study observed that Hispanics and non-Hispanic blacks were more likely to have ER-positive and PR-negative tumours than non-Hispanic whites [30]. However, in our study, we found no interaction between subtype and race.

The protective effect of marriage on survival can be explained by these patients gaining better economic resources and having greater social support in marriage [31]. Although some factors have been found in previous studies, no covariates have been adjusted for other factors, or fewer covariates have been adjusted. We used a Cox proportional hazards regression model adjusting for all the factors, which demonstrated that tumour subtype was a prognostic factor. Therefore, in clinical and nursing work, doctors and nurses can carry out different treatments and nursing work for different patients according to age, race, marital status, insurance, tumour grade, histology, subtype, and visceral metastases. In addition, we found that there was an interaction between subtype and multiple visceral metastases, which suggests that we should pay attention to the risk of visceral metastasis in patients with different subtypes. Future studies are recommended to explore the mechanism of molecular subtype and metastasis site as well as the influence of their interaction on the outcome and management of patients.

We acknowledge that the study has some limitations. The SEER database does not provide information on the expression status of Ki-67; the Ki-67 index value is a prognostic factor in primary breast cancer and is a proliferation marker that also distinguishes between luminal A and luminal B breast cancer [32]. Breast cancer is generally divided into luminal A and luminal B according to HR/HER2 status and Ki-67 in the course of clinical diagnosis and treatment [33]. This may contribute to some disparities between our investigation and clinical applications. We do not have information regarding the radiotherapy or systemic treatments of this cohort, which may contribute to some of the differences observed in survival according to prognostic variables. Additionally, the pathological data could not be centrally reviewed and were collected from different local pathology laboratories.

## Conclusion

In conclusion, our results revealed a relatively good prognosis for stage IV patients with bone metastasis. The median OS was 38 months, and 33.9% of stage IV patients were alive at 5 years. Subtype was a significant prognostic factor, and the prognosis of stage IV patients with the HR−/HER2- subtype was the worst, with a median OS of only 11 months. In addition to tumour subtype, race, marital status, insurance, grade, site of metastases, and surgery were independent predictors of OS. There was an interaction between subtype and multiple visceral metastases.

## Data Availability

The datasets analyzed during the current study are available in the SEER repository (https://seer.cancer.gov/). The databases are public access.

## Abbreviations

OS: Overall survival
BCSS: Time from breast cancer diagnosis to death due to breast cancer
ER: Oestrogen receptor
PR: Progesterone receptor
HR-: ER- and PR-
HR+: ER+ and/or PR+
HR: Hazard ratio
HER2: Human epidermal growth factor receptor-2
CI: Confidence interval
ICD-O-3: International Classification of Diseases for Oncology, 3rd Edition
